# Hemoglobin-based oxygen carriers: evolution, translational applications and clinical considerations

**DOI:** 10.3389/fmed.2026.1939029

**Published:** 2026-10-05

**Authors:** Huan Tang, Xinyi Liu, Hai Zhang, Xiaobo Huang, Chun Pan, Lin Chen

**Affiliations:** 1 North Sichuan Medical College, Nanchong, China; 2 Centre for Critical Care Medicine, Sichuan Provincial People’s Hospital, University of Electronic Science and Technology, Chengdu, China; 3 Department of Geriatric Internal Medicine, Sichuan Provincial People’s Hospital, University of Electronic Science and Technology, Chengdu, China

**Keywords:** biological engineering, hemoglobin oxygen carrier, oxidative stress, oxygen nanocarriers, oxygen-carrying capacities, translational medicine

## Abstract

Tissue survival depends on adequate oxygen delivery, yet when red blood cell (RBC) transfusions are unavailable or contraindicated, restoring oxygen delivery remains an urgent clinical need. To meet this need, researchers have developed hemoglobin-based oxygen carriers (HBOCs) that transport oxygen via cell-free hemoglobin circulating in plasma. However, the intrinsic toxicity of cell-free hemoglobin (Hb) has slowed its clinical translation. More fundamentally, it has shifted the field's focus from universal blood substitutes toward indication-specific solutions. Despite persistent mitigation efforts, no HBOC has gained full Food and Drug Administration (FDA) approval for human use, suggesting that these toxicities are not tractable engineering problems but intrinsic liabilities of the cell-free Hb molecule. A meta-analysis of early clinical trials reported increased risks of death and myocardial infarction with several HBOCs, prompting a clinical hold on HBOC trials in the United States. In this review, we trace the evolution of engineering strategies and examine the functional mechanisms that underpin these approaches. We then evaluate HBOCs not as universal RBC substitutes but as context-dependent strategies for oxygen delivery across acute resuscitation, organ preservation, and hypoxia-driven pathologies, followed by a safety assessment. In each scenario, clinical value rests on providing temporary oxygen support when standard transfusion is unavailable or inadequate, rather than on replicating the full functionality of RBCs. Although a universal RBC substitute remains a distant goal, HBOCs are progressively evolving into oxygen therapeutics specific to indications. Their clinical adoption will require rigorous evidence of long-term safety, manufacturing reproducibility, and clinically meaningful efficacy in well-defined patient populations.

## 1 Introduction

Adequate oxygen supply is the prerequisite for cell metabolism and organ function (1). In vertebrates, red blood cells (RBCs) serve as the main carriers of oxygen throughout the body (2). Within RBCs, hemoglobin (Hb), a tetrameric heme protein whose ferrous iron (Fe²⁺) reversibly binds oxygen, is packed at high intracellular concentration and thereby provides a large oxygen-carrying capacity. Its cooperative binding and allosteric regulation enable efficient oxygen loading in the lungs and controlled release in the microcirculation (3). When the total number of RBCs in circulation is seriously insufficient, restoring oxygen-carrying capacity through allogeneic blood transfusion remains a life-saving intervention (4).

Therefore, blood transfusion is an essential therapeutic intervention in contemporary clinical practice. However, its availability is often constrained by donor shortages, limited storage duration, cold-chain requirements, blood-group compatibility (5). Transfusion also carries risks including transfusion-related complications (6). These limitations are particularly pronounced during mass-casualty incidents, military operations, natural disasters, pandemics, as well as in remote or resource-limited environments, where timely access to compatible blood products is frequently compromised (7, 8).

These challenges have drawn sustained attention to alternative strategies to maintain tissue oxygenation when RBC transfusion is impossible (9). Among these, hemoglobin-based oxygen carriers (HBOCs) have been extensively researched because Hb, as a natural oxygen binding protein in RBCs, can facilitate immediate oxygen transport and is not limited by blood type matching or the storage limitations of donor blood (10). However, a meta-analysis of 16 randomized trials reported increased risks of death and myocardial infarction with several HBOCs (11), and no HBOC has since gained the Food and Drug Administration (FDA) approval for human use (12). Early cell-free Hb preparations and a number of chemically modified products were associated with vasoconstriction, hypertension, and oxidative tissue damage (13). These adverse reactions mainly arise from the ability of extracellular Hb to remove nitric oxide (NO), undergo autoxidation, generate methemoglobin (MetHb), and destroy endothelial homeostasis (14–16). These findings have shifted the field's research from empirical Hb stabilization to mechanism-based design. This approach integrates protein engineering, polymer chemistry and materials science to improve both oxygen supply capacity and biocompatibility simultaneously.

Various chemical and macromolecular domains, including polymer coupling, polymerization, and cross-linking, have been used to increase the molecular size of HBOCs. These approaches have been implemented to prolong the circulatory half-life and reduce renal clearance of HBOCs. Surface engineering methods, including polydopamine coatings and metal-phenolic networks (MPNs), have been investigated for their potential to impart antioxidant and endothelial-protective properties (17, 18). At the molecular level, rational protein design has also been applied. A notable example is the β-F41K mutation (β-chain phenylalanine-to-lysine substitution at position 41), designed to stabilize the heme microenvironment, preserve redox homeostasis, and limit cofactor loss (19). Concurrently, nanostructured encapsulation systems, such as zeolitic imidazolate framework-8 (ZIF-8) scaffolds, lipid vesicles, and polymeric microgels, generate biomimetic microenvironments that potentially enhance Hb stability, restrict direct plasma exposure, and facilitate more tightly regulated oxygen release (20–22). Collectively, these strategies represent a shift from chemically stabilized Hb solutions toward more physiologically integrated oxygen-delivery systems.

The level of translational evidence varies widely among HBOC platforms. Cross-linked and polymerized Hb (e.g., diaspirin cross-linked and glutaraldehyde-polymerized products) have reached phase II/III clinical trials (23, 24), as have selected PEGylated Hb products (25). In contrast, most encapsulation-based systems, including liposomal, microgel, and framework-based carriers, have only undergone *in vitro* characterization and preclinical testing (26). Therefore, it is essential to distinguish among these levels of evidence, which include clinically validated efficacy, *in vivo* data, experimentally confirmed mechanisms, and unverified applications.

Although there have been several reviews of the engineering design of HBOCs (27–29), most of the existing studies discuss a single platform in isolation, rather than incorporating it into a maturity-based framework for systematic synthesis. In this review, we divide HBOC-related strategies into three levels: (i) products that have entered early clinical trials or obtained compassionate use authorization; (ii) preclinical research platforms that have passed *in vivo* proof of concept; and (iii) exploratory biological materials at the *in vitro* characterization stage. For each level, we evaluate the safety and efficacy of different design strategies. Here, efficacy is defined according to the evidentiary tier: clinical outcomes for products in clinical trials, *in vivo* oxygen-delivery performance for preclinical platforms, and *in vitro* oxygen-binding and release properties for exploratory materials. We further weigh the proposed mechanisms according to the rigor of the supporting evidence, to distinguish between the function that has been clinically verified and the function that is still in the experimental research stage.

We propose the concept of the “oxygen bridge”, a carrier that temporarily sustains tissue oxygenation when the body's own oxygen-carrying capacity is impaired, and with a role that varies across clinical contexts. Our concern is not whether these oxygen carrier preparations can replicate the function of RBCs, but whether they can provide effective temporary oxygen supply when blood transfusion is unavailable or inadequate.

## 2 Literature search strategy

This narrative review was conducted using a structured search of PubMed, Web of Science, Scopus, and Google Scholar, for the period from January 2000 to December 2025; the search was last updated in February 2026. Landmark studies published before 2000 were included if they provided foundational clinical evidence. The search strategy was designed to cover the following areas: the concept of blood substitutes, toxicity mechanisms, translational applications, and the historical development of HBOCs. Disagreements regarding inclusion criteria or evidence classification were discussed until consensus was reached. Non-English literature, conference abstracts lacking sufficient methodological detail, editorials, and research not directly related to HBOC formulation, function, safety, or clinical translation were excluded from the analysis.

## 3 The evolution and historical development of HBOCs

### 3.1 Overview of historical evolution

Research on artificial blood substitutes can be traced back to the early 20th century and was driven by the urgent need for alternatives to donated blood. Typical application scenarios include trauma resuscitation and combat casualty care (30). From this need, two main technical routes emerged. Early research focused on perfluorocarbon (PFC) emulsions, which carry oxygen through physical dissolution. Subsequent research focused on HBOCs, which are designed to mimic the oxygen-binding and release characteristics of natural RBCs (31–33). Although PFC emulsions have high oxygen solubility, their volatility, emulsion instability, and limited biocompatibility have hindered their widespread clinical application (34). 

The first generation of HBOCs was composed of chemically stabilized cell-free Hb. Although these compounds entered the clinical trial, their application was eventually limited by toxicity, mainly due to NO scavenging and oxidative instability (23, 35, 36). These findings prompted researchers to shift their focus from empirical chemical modification toward mechanism-based design strategies. In these mechanistic studies, molecular size was a key parameter, because smaller Hb species can cross the vascular endothelium and scavenge NO (37). Unmodified cell-free Hb (∼64 kDa) dissociates rapidly into αβ dimers and is cleared by the kidneys, resulting in a very short circulatory half-life. Its small size allows Hb to enter the the subendothelial space, where it can scavenge NO and cause vasoconstriction (38). To address these limitations, subsequent HBOC designs employed polymer chemistry, protein engineering, and nanoscale encapsulation technology (18, 20). These modifications increased the hydrodynamic radius and molecular weight of the constructs, thereby prolonging intravascular residence time and reducing endothelial extravasation. However, merely increasing molecular size did not eliminate HBOC-associated toxicity. The degree to which these strategies mitigate NO scavenging, oxidative stress, and resulting vascular side effects; further preclinical and clinical validation is therefore needed (39). Therefore, molecular size should be considered part of a broader safety and efficacy strategy rather than a standalone solution.

### 3.2 First-generation HBOCs

Early research began with stroma-free Hb (SFH), prepared by removing RBC membranes from expired human or bovine blood to eliminate blood group antigens. However, unmodified SFH is structurally unstable: outside the protective environment of RBCs, the Hb tetramer (α_2_β_2_, ∼64.5 kDa) spontaneously dissociate into αβ dimers (∼32 kDa), which are readily filtered through the glomerular filtration barrier, resulting in heme nephrotoxicity (40). To stabilize the tetramer, researchers developed a chemical cross-linking strategy to extend the intravascular residence time of Hb (41–43). The most widely studied of these was diaspirin cross-linked Hb (DCLHb, also known as HemAssist), in which the two α-globin subunits are covalently cross-linked with bis(3,5-dibromosalicyl) fumarate to prevent dissociation into dimers. However, multicenter randomized trials indicated a higher mortality rate in patients treated with DCLHb compared with those receiving standard resuscitation fluids (44). In a randomized controlled trial published in the Journal of the American Medical Association (JAMA), Sloan et al. (44) found that 24 of 52 patients (46%) who received DCLHb died within 28 days, vs. 8 of 46 patients (17%) who received saline (*p* = 0.003). The authors concluded that DCLHb did not appear to be an effective resuscitation fluid. Notably, a separate analysis by the same group found that DCLHb treatment was not associated with base deficit or lactate abnormalities, suggesting that the increased mortality could not be explained by impaired tissue perfusion alone. The failure of DCLHb prompted a systematic investigation into the mechanisms of HBOC toxicity.

Subsequent studies identified two principal toxicity pathways. First, cell-free Hb scavenges NO efficiently, resulting in reduced NO bioavailability within endothelial cells, which has been associated with systemic and pulmonary hypertension (13). Second, the Fe^2+^ of Hb is spontaneously oxidized to a ferric (Fe^3+^) state, thereby producing superoxide anions and hydrogen peroxide. These reactive oxygen species (ROS) aggravate oxidative stress, promote lipid peroxidation, and activate pro-inflammatory signaling pathways (45, 46).

In summary, the clinical experience of the first-generation HBOCs shows that only enhancing the oxygen-carrying capacity of plasma is not enough to overcome the vasoactivity and inadequate safety. After researchers realized that vascular toxicity and oxidative stress posed more urgent obstacles to clinical translation than inadequate oxygen delivery, the research focus gradually shifted to rational design at the molecular level.

### 3.3 Second-generation and next-generation HBOCs

First-generation products had limitations such as nephrotoxicity and heme redox reactivity (47, 48). In response, second-generation strategies shifted from empirical chemical modification to rational molecular engineering, using recombinant biotechnology, polymer coupling, and nanoencapsulation to improve circulatory stability, vascular compatibility, and oxygen-carrying capacity (26, 49).

Several second-generation products based on polymerization entered clinical evaluation. PolyHeme, a polymerized human Hb formulation, was developed for prehospital resuscitation of hemorrhagic shock when blood is unavailable. An early small trial suggested safety and reduced allogeneic blood use (36). However, a subsequent multicenter phase III trial in 714 patients found no significant difference in 30-day mortality between the PolyHeme and control groups (13.4% vs. 9.6%). Adverse events were more frequent in the PolyHeme group (93% vs. 88%, *p* = 0.04), casting doubt on its clinical utility even in settings where blood is unavailable (50).

HBOC-201 [Hb glutamer-250 (bovine), trade name Hemopure], a glutaraldehyde-polymerized bovine Hb (PolybHb), has received limited regulatory approval in South Africa and Russia (10). However, evidence on HBOC safety varies across clinical settings. In the HEM-0115 phase III trial of elective orthopedic surgery, patients who received HBOC-201 showed no significant difference in mortality compared with those who received erythrocytes (15). However, a re-analysis of this trial reported a higher incidence of cardiac serious adverse events (6.3% vs. 2.7%, *p* ≤ 0.05) and troponin elevations (13.2% vs. 1.6%, *p* = 0.0003) with HBOC-201 than with RBCs, although these findings were not accompanied by a significant increase in acute coronary syndrome or mortality (51). An analysis of 1,701 clinical uses found that HBOC-201 was well tolerated in a wide range of doses and clinical settings, with no evidence of cardiotoxicity. Common adverse reactions include transient hypertension, oliguria, and gastrointestinal symptoms (52). A separate phase III trial in noncardiac surgery reported transient hypertension, with no statistically significant increase in clinically evident cardiac adverse events. However, cardiac biomarkers were not systematically measured, and the small sample size limited the detection of subclinical myocardial injury and rare cardiac events (53). Collectively, these findings indicate that the cardiac safety of HBOC-201 remains inconsistent, which is a major limitation to the use of these agents as an alternative to RBCs. Rather than being confined to HBOC-201, cardiac and mortality signals have been apparent across the class. A 2008 meta-analysis of 16 randomized trials (3,711 patients) spanning five cell-free Hb products reported increased risks of death [risk ratio (RR), 1.30; 95% CI, 1.05–1.61] and myocardial infarction (RR, 2.71; 95% CI, 1.67–4.40), with no indication that the excess risk was limited to a particular product or indication (11), although the appropriateness of pooling pharmacologically distinct compounds was subsequently debated (12). In response to concerns raised by this meta-analysis, the FDA placed a clinical hold on HBOC trials in the United States in 2008 (12). At a public workshop convened by the FDA and the National Institutes of Health in April 2008, Natanson concluded that further trials should not proceed until the mechanisms of toxicity had been elucidated and corrected (47), and argued that any existing or developing HBOC should first undergo preclinical animal testing for known toxicities, with complete and timely disclosure of trial data (11). Chen et al. recommended that such studies employ prespecified endpoints, independent investigators, and FDA oversight (54). Building on these recommendations, new-generation carriers should proceed cautiously through a stepwise safety pipeline, from molecular and vascular characterization (e.g., NO reactivity, autoxidation, and residual tetramer content, together with endothelial-injury and vasoactivity assays) to human-derived model systems, including endothelial and cardiac organoids, to conscious-animal studies of hemodynamics and myocardial injury, before any early-phase human study is considered.

These clinical advances have prompted researchers to study Hb in greater depth. An important advance in Hb engineering is the application of recombinant DNA technology and site-directed mutagenesis to modify amino acid residues near the heme pocket, thereby modulating oxygen affinity and suppressing autoxidation. Representative examples include engineered Hb variants, such as those involving β93Cys (the cysteine residue at position 93 of the β-globin chain) and rHb1.1 (a recombinant Hb variant), which improve oxidative stability through the formation of specific disulfide bonds (55, 56). Nevertheless, impurities, endotoxin control, protein folding, heme binding, and quality production that meets regulatory standards remain challenging. To maintain functional integrity, proper heme incorporation and binding are still needed to ensure correct protein folding and the formation of active sites (57).

In parallel with protein engineering of Hb, polymer coupling and surface shielding strategies have become important engineering methods. Among them, PEGylated Hb (PEG-Hb) represents a remarkable progress. Conjugation with polyethylene glycol (PEG) can increase the hydrodynamic radius, limit Hb extravasation and its interaction with endothelial cells, and reduce adhesion based on non-specific charges by shielding surface residues (58). In the preclinical model, PEG-Hb showed a longer circulatory half-life, lower NO scavenging activity, and weakened hypertensive reaction (59). PEG-modified HBOC formulations, such as SANGUINATE® (PEGylated bovine carboxy Hb) and MP4OX® (maleimide-PEGylated human Hb, formerly Hemospan), have advanced to Phase I and II clinical trials, during which their safety, tolerance, and endpoint measures related to oxygen supply have been evaluated. However, consistent evidence of clinical benefit remains limited and depends on the specific indication (60).

Beyond surface modification, nanoencapsulation represents a more radical engineering strategy. In this approach, Hb is encapsulated within liposomes or other vesicle nanocarriers to form Hb vesicles (HbVs) or liposome-encapsulated Hb (LEH) (61).  This structure forms a physical barrier between Hb and plasma components, thereby reducing direct interaction with vascular endothelium and partially mimicking the biochemical microenvironment of RBCs. Sakai et al. (62) demonstrated that in the preclinical models, HbVs can attenuate vascular reactivity and reduce oxidative stress while maintaining oxygen transport efficiency.  In addition, these encapsulated systems exhibit high physical and chemical stability and can be engineered to integrate antioxidant enzymes or molecular sensors that regulate ROS generation (63). Therefore, encapsulated HBOCs represent a new class of oxygen carriers that can be experimentally modulated and possess biomimetic properties. However, their clinical efficacy has yet to be validated. Table 1 provides a comparative overview of the major HBOC platforms, summarizing representative systems, design concepts, O_2_-delivery rationale, major limitations, and development status. Two conclusions can be drawn from this comparison. First, NO scavenging, vasoconstriction, and oxidative stress persist across the cell-free Hb platforms, albeit to varying degrees, confirming that these toxicities are intrinsic to acellular Hb. By contrast, encapsulation-based carriers (e.g., HbVs) mitigate these effects through physical shielding but face distinct challenges such as mononuclear phagocyte system (MPS) uptake and complement activation. Second, the gap between preclinical promise and clinical outcomes is a recurring theme. Platforms that perform well in animal models, such as HbVs and recombinant Hb, have yet to produce definitive human efficacy data.

**Table 1:** Comparative overview of major HBOC platforms and engineering strategies.

Platform (engineering approach)	Representative systems	Design concept	O_2_ rationale/advantages	Major limitations	Status	Ref.
Stroma-free Hb	Early cell-free Hb	Purified acellular Hb	Direct plasma O_2_ delivery	Rapid renal filtration; short half-life; NO scavenging; vasoconstriction; oxidative injury	Abandoned	(13)
Cross-linked Hb	DCLHb (HemAssist)	Intramolecular cross-linking stabilizes the tetramer	Reduced dissociation; prolonged retention	Increased mortality in a trauma trial; persistent NO scavenging; hypertension; MetHb risk	Discontinued	(23, 35, 36)
Polymerized Hb	HBOC-201 (Hemopure); PolyHeme	Polymerization increases size and reduces renal loss	Prolonged retention; functional O_2_ delivery	Hypertension; oxidative stress; cardiovascular safety concerns	Hemopure: limited approval in South Africa; PolyHeme: discontinued	(11, 64)
PEGylated Hb	MP4OX; SANGUINATE (PEG-carboxyHb)	PEG conjugation increases hydrodynamic size	Increased persistence; microvascular delivery; CO-mediated cytoprotection (SANGUINATE)	Residual NO/oxidative/MetHb risks; potential PEG immunogenicity	Clinical development; limited translation	(25, 65, 66)
Lipid-vesicle carriers	HbV; LEH	Liposomal encapsulation provides RBC-mimetic shielding	Reduced plasma interactions; controlled O_2_ release	MPS uptake; complement activation; lipid oxidation; scale-up	Preclinical	(67, 68)
MOF-based carriers	Hb@MOF; Hb@ZIF-8	Hb immobilization or encapsulation in porous MOFs	Potential controlled release and catalytic/antioxidant functions	Metal-ion release; uncertain biodegradation and clearance	Proof of concept	(69, 70)
Polymeric/protein-based carriers	Polymeric nanoparticles; albumin-based carriers; nanogels	Hb encapsulation, loading, or conjugation	Hb shielding; potentially controlled O_2_ delivery	MPS uptake; biocompatibility; clearance and scale-up	Preclinical	(71, 72)
Marine extracellular Hb	M101 (HEMO2Life®)	Extracellular Hb isolated from Arenicola marina	High O_2_-carrying capacity; broad pH tolerance; reduced NO reactivity; high thermostability	Potential xenogeneic immunogenicity; not established for systemic infusion	Organ-preservation use/evaluation	(73, 74)
Recombinant/genetically engineered Hb	Recombinant Hb variants; fusion mutants	Sequence engineering modifies O_2_, redox, and NO properties	Tunable O_2_ affinity; potentially reduced NO scavenging	Production cost/yield; redox instability; regulatory complexity	Preclinical/early translational	(49, 75)

HBOC, hemoglobin-based oxygen carrier; Hb, hemoglobin; O_2_, oxygen; NO, nitric oxide; MetHb, methemoglobin; CO, carbon monoxide; PEG, polyethylene glycol; MOF, metal–organic framework; ZIF, zeolitic imidazolate framework; MPS, mononuclear phagocyte system; RBC, red blood cell.

## 4 Molecular and engineering strategies for next-generation HBOCs

Early HBOCs had limited success, which shifted research from empirical chemical modification to rational molecular design. Current work draws on polymer chemistry, protein engineering, nanotechnology, and biomaterials science to develop more biocompatible and controllable oxygen-delivery platforms. Most of these platforms are still in preclinical or early translational stages (29, 76, 77). Traditional methods focused on molecular volume or circulation half-life. In contrast, modern strategies seek to improve how Hb functions within its biological microenvironment. These strategies aim to reduce NO scavenging, strengthen redox homeostasis, and improve microvascular compatibility. These objectives are addressed through four distinct molecular engineering strategies, summarized in Figure 1.

**Figure 1:**
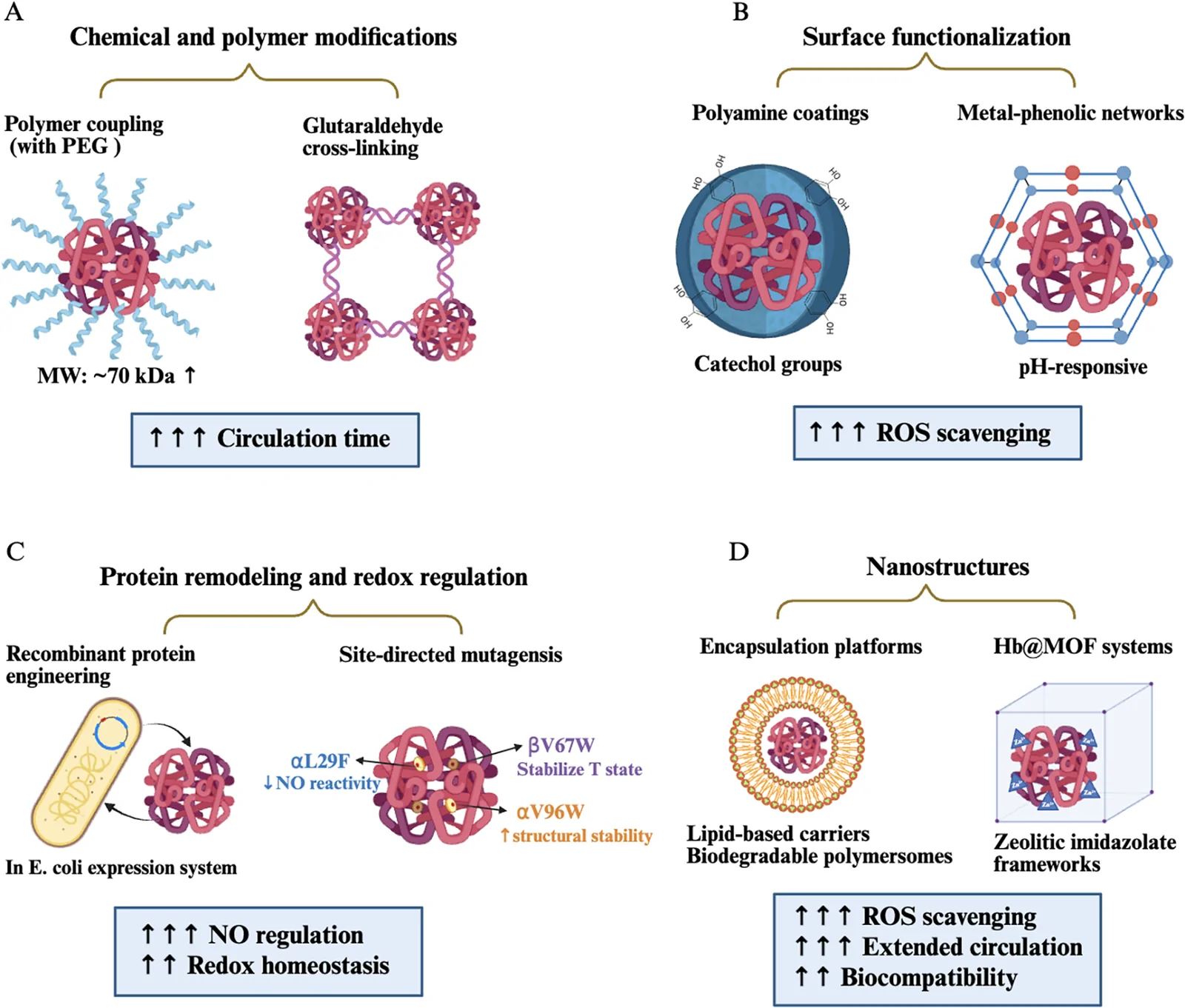
Molecular engineering strategies for next-generation HBOCs. Four complementary approaches to improve HBOC performance: chemical/polymer modifications extend circulation; surface functionalization enhances ROS scavenging; protein remodeling improves NO regulation; nanostructured systems provide multifunctional enzymatic protection.

### 4.1 Chemical and polymer modifications

Polymer coupling is an engineering strategy for stabilizing free Hb and regulating the oxygen partial pressure at which Hb is 50% saturated (P_50_). Conjugation with high-molecular-weight PEG increases the hydrodynamic radius, which can reduce renal filtration and attenuate NO scavenging. PEG-based formulations derived from bovine and human Hb, such as SANGUINATE and MP4OX, have progressed to clinical trials, where they have been evaluated for circulatory stability, hemodynamic tolerability, and plasma retention (78). McConachie et al. (79) reported three patients with severe anemia who declined blood transfusion for religious reasons and received SANGUINATE on an emergency basis. Of the three patients, one recovered and two died. A phase II trial in 90 hip surgery patients showed that MP4OX reduced the incidence of hypotension by nearly half compared with Ringer's acetate (a balanced crystalloid) infusion (*p* < 0.001). However, the relevance of these findings to future clinical use remains unclear, particularly in high-risk patients with ischemia and oxygen debt (80).

Polymerization of Hb with a bifunctional reagent such as glutaraldehyde is controlled by fixing the cross-linker-to-Hb ratio and by quenching the reaction to a predetermined extent. Glutaraldehyde forms intramolecular cross-links that stabilize the tetramer and intermolecular cross-links that join tetramers into larger polymers, so the degree of cross-linking largely determines the average molecular weight (81). Because cross-linking is a stochastic process, the initial product is polydisperse. Size fractionation by size-exclusion chromatography or tangential flow filtration removes unpolymerized and oversized species, yielding a narrower molecular weight distribution (82). This more uniform size distribution allows more precise tuning of viscosity and, consequently, of the oxygen transfer kinetics of the product (83). Other polymer coupling strategies, including covalent conjugation with dextran or albumin, have shown potential to enhance colloidal stability and reduce immunogenicity in experimental studies (84, 85). However, for PEGylated Hb, although the dense PEG shell may improve vascular compatibility, excessive surface shielding reduces shear-dependent oxygen release, a trade-off that remains controversial in hemodynamic modeling.

### 4.2 Surface functionalization

In addition to polymer coupling, surface-functionalized HBOCs offer enhanced control over biological interactions, enabling modulation of oxidative stress, inflammatory responses, and oxygen release profiles. Polydopamine (PDA) coatings can form an antioxidant barrier rich in catechol groups, and experimental models have confirmed that these groups can neutralize ROS (86). MPNs composed of tannic acid and Hb have also been studied as a platform for the reversible storage and release of NO to maintain vascular homeostasis (87). In addition, adaptive coatings responsive to changes in pH or redox state are currently being studied to achieve controlled oxygen release in hypoxic microenvironments (88). These surface engineering methods are expected to transform HBOCs from passive oxygen carriers into an adjustable oxygen therapy platform. However, these observations should be interpreted with caution (89). First, most “smart” surface modifications, such as pH-responsive or redox-responsive coatings, have been tested only in defined buffer systems or static cell cultures. These conditions lack the spatiotemporal complexity of *in vivo* oxidative and pH gradients. Second, most Hb modifications have not been evaluated in large animal models of hemorrhage or ischemia-reperfusion using clinically meaningful endpoints.

### 4.3 Protein remodeling and redox regulation

Recombinant protein engineering tailors Hb properties by introducing defined changes into the globin genes, which are then expressed in recombinant *Escherichia coli*. The approach relies on two complementary strategies: site-directed mutagenesis of individual residues and genetic fusion of the subunit genes. Fusion of the two α-subunit genes through a short glycine linker covalently joins the α subunits, thereby preventing the tetramer from splitting into αβ dimers that are rapidly cleared by the kidney (90). Site-directed mutagenesis tunes NO reactivity and O_2_ affinity: substitutions in the distal heme pocket (αL29F, βV67W) reduce the rate of NO deoxygenation (64), while mutations at the allosteric interface (αV96W) stabilize the T state and raise P_50_ (91). Such designs offer precise control over ligand binding and heme stability *in vitro*, yet translating them into clinical applications remains difficult. The β-F41K mutation illustrates this *in vitro*–*in vivo* gap: it significantly reduces heme dissociation and auto-oxidation, thereby enhancing heme retention and thermal stability *in vitro* (19). It is not yet known whether this stabilization persists under the oxidative and proteolytic conditions of circulating plasma *in vivo*. In addition, a potential but unexplored strategy to maintain Hb in its functional ferrous state would be to co-express two oxidoreductases: cytochrome b5 reductase, which reduces MetHb to ferrous Hb, and catalase, which scavenges H_2_O_2_.

More fundamentally, recombinant HBOCs face persistent manufacturing challenges (92). These include low expression yields, complex and costly purification, and batch-to-batch variability that complicates regulatory approval. The field must carefully assess whether the advantages of recombinant approaches justify their added manufacturing complexity relative to chemically modified natural Hb.

### 4.4 Nanostructures and hybrid packaging systems

Encapsulation of Hb in a nano- or micron-level carrier can partially reproduce the specific topology and barrier characteristics of RBCs, and is expected to reduce MetHb formation. By co-encapsulating antioxidant enzymes such as superoxide dismutase (SOD) and catalase, LEH and HbV platforms aim to partially recapitulate the antioxidant defense system of RBCs; these constructs are currently being evaluated in preclinical models (63, 93). Azuma et al. (93) conducted a phase I trial of HbVs as a transfusion alternative. Adverse events were mild and self-limiting. Based on these results, a phase II dose-escalation trial with a larger sample size is planned. Yadav et al. (94) found that resuscitation with nanoparticulate LEH creates a pro-survival phenotype in hemorrhaged rats. Owing to its oxygen-carrying capacity, LEH performs significantly better than saline in hemorrhagic shock. In parallel, biodegradable amphiphilic copolymers that assemble into micelles and vesicles have been explored to encapsulate or conjugate Hb as degradable artificial RBCs, a strategy reviewed by Li et al. (95). In a representative example, a triblock copolymer of poly(ethylene glycol) and a biodegradable polyester formed asymmetric polymersomes that entrapped Hb without damaging its bioactivity; the resulting vesicles showed oxygen affinity close to that of native Hb (P_50_ ≈ 21.6 mmHg vs. 22 mmHg) and, combined with a plasma expander, reduced early mortality and prevented acute tubular necrosis in an acute anemia rat model (96). More recently, two graft copolymers with reversed hydrophilic and hydrophobic segments assembled cooperatively into compact vesicles of about 170 nm at a high hydrophilic fraction of about 60%, which resisted protein adsorption and prolonged blood circulation while preserving the oxygen binding activity of encapsulated Hb (P_50_ ≈ 29.5 mmHg vs. 27 mmHg for native Hb) (97).

Emerging hybrid architectures based on Hb@metal–organic frameworks (Hb@MOFs), including zeolitic imidazolate frameworks (ZIFs) such as Hb@ZIF-8, use porous scaffolds to limit ROS ingress while promoting oxygen diffusion (70, 98). In parallel, extracellular Hb M101 (trade name HEMO2Life®, derived from the marine worm *Arenicola marina*) has attracted wide attention for its broad pH tolerance, minimal NO reactivity, and high thermostability in both experimental and organ-preservation studies (74, 99). PEG-M101 have further demonstrated improved oxygen-carrying capacity and enzymatic resilience in organ perfusion models (100). Together, lipid-based carriers, biodegradable polymeric assemblies, and inorganic or protein-derived hybrid systems represent complementary strategies for enhancing carrier stability and functionality. Nevertheless, most of these systems remain confined to preclinical or organ-preservation applications, and their translational path toward therapeutic HBOCs requires rigorous *in vivo* validation.

## 5 Functional mechanisms underlying HBOC performance

The physiological effects of HBOCs depend on four interrelated mechanisms: oxygen binding and release, redox balance, vasoactivity, and immune compatibility (Figure 2) (76, 92, 101).

**Figure 2:**
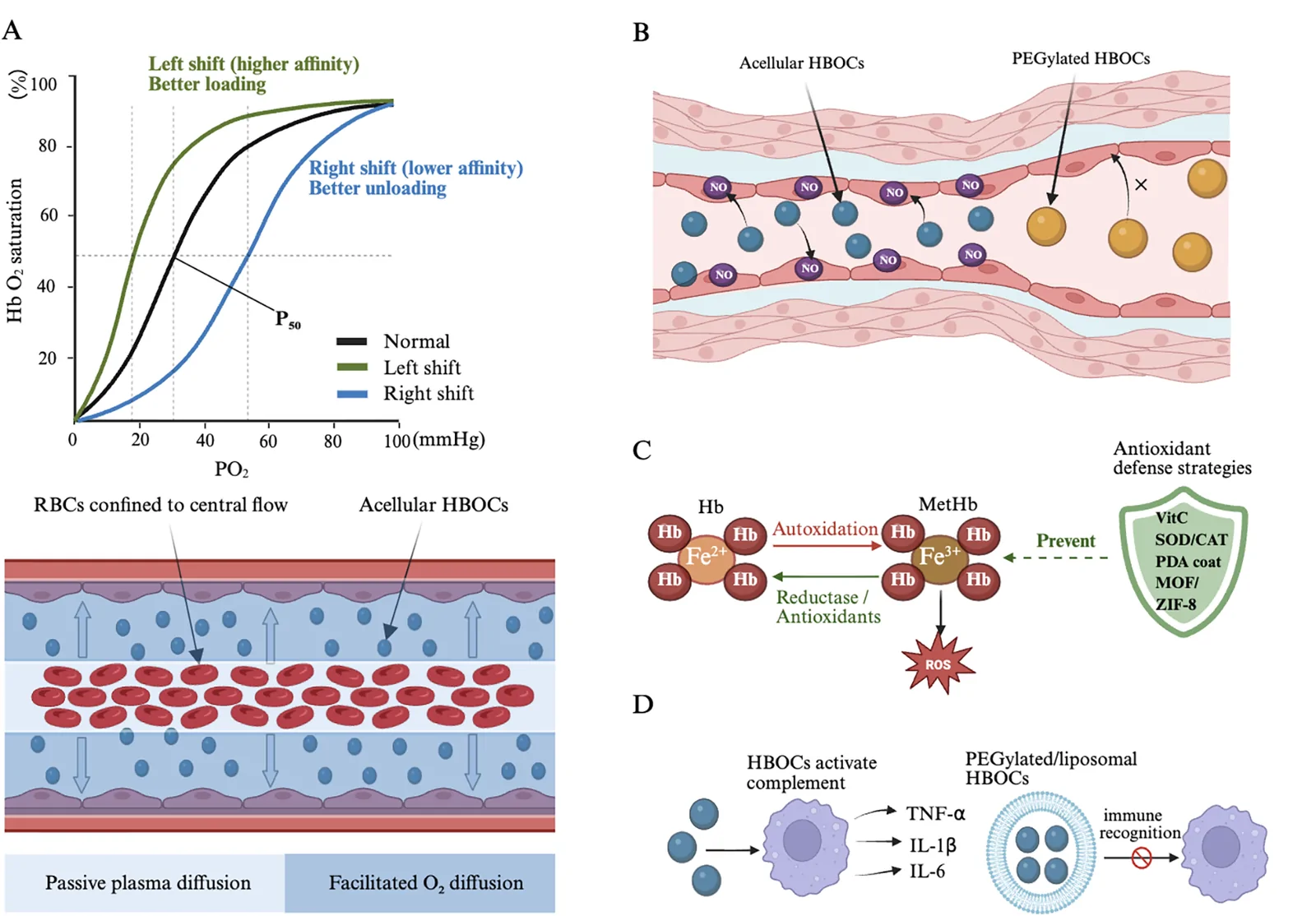
Functional mechanisms and safety challenges of HBOCs. **(A)** (Top) Oxygen dissociation curves: RBCs serve as the reference; high-affinity HBOCs are left-shifted (lower P_50_), favoring O_2_ loading in the lungs; low-affinity HBOCs are right-shifted (higher P_50_), favoring O_2_ unloading in tissues. (Bottom) Microvascular transport: RBCs are confined to the axial flow due to their size; HBOCs enter the cell-free layer, shorten the O_2_ diffusion distance, and enable facilitated O_2_ diffusion. Together, the two subpanels show that HBOCs enhance tissue oxygenation through tunable O_2_ affinity and a shorter O_2_ diffusion distance. **(B)** Small, unmodified HBOCs cross endothelial gaps, scavenge NO, and cause vasoconstriction and hypertension. Surface modifications such as PEGylation increase molecular size, prevent extravasation, and preserve NO bioavailability. **(C)** Hb-Fe^2+^ autoxidizes to MetHb-Fe³⁺, loses O_2_-carrying capacity, and generates ROS. This can be mitigated by antioxidant strategies. **(D)** Unmodified HBOCs activate the complement system and induce macrophages to release proinflammatory cytokines. PEGylation or liposomal encapsulation can shield them from immune recognition and prolong circulation time.

### 5.1 Kinetics of oxygen binding and release

The efficiency of oxygen loading and unloading in HBOCs is mainly determined by two intrinsic properties: oxygen affinity (represented by P_50_) and cooperative oxygen binding (reflected by the Hill coefficient, a measure of binding cooperativity) (102, 103). Compared with native RBCs, modified HBOCs usually exhibit a slightly higher P_50_ value, which facilitates oxygen release at the tissue level. The optimal oxygen affinity is highly context-dependent. For example, during organ preservation, enhanced oxygen retention (i.e., a leftward shift of the oxygen dissociation curve) is preferable, whereas in trauma resuscitation, a rightward shift is preferred to promote rapid oxygen release (104). Although the theoretical importance of P_50_ has been established, there is little clinical evidence that modulating the oxygen affinity of HBOCs improves patient outcomes. The two HBOCs most commonly studied in clinical trials are HBOC-201 (P_50_ ≈ 38 mmHg) (105) and DCLHb (P_50_ ≈ 32 mmHg) (106). Although their oxygen affinities are similar, their safety profiles differ markedly. This finding indicates that clinical outcomes cannot be accurately predicted by P_50_ alone and that efficacy evaluation criteria beyond oxygen affinity are needed.

The Hill coefficient (*n*_H_), the second intrinsic property, quantifies the cooperativity of Hb–O_2_ binding and thereby the steepness of the oxygen dissociation curve. *n*_H_ is obtained as the maximal slope of a Hill plot of log [*Y*/(1 − *Y*)] vs. log PO_2_, where *Y* is the fractional O_2_ saturation (107). It is an empirical descriptor rather than a true molecular constant, with an upper limit equal to the number of binding sites (four for tetrameric Hb). Three cases are conventionally distinguished: *n*_H_ = 1 denotes non-cooperative (hyperbolic) binding, characteristic of monomeric globins such as myoglobin; *n*_H_ > 1 denotes positive cooperativity, which produces the sigmoidal curve that allows native Hb to respond sharply to small changes in PO_2_; and *n*_H_ < 1 reflects negative cooperativity or heterogeneous binding sites. The value *n*_H_ of ≈2.8–3.0, measured for native human Hb under standard conditions (pH: 7.4, 37 °C) therefore indicates strong but incomplete cooperativity. This cooperativity arises from the allosteric T → R quaternary transition, in which O_2_ binding at one subunit increases the affinity of the remaining subunits (108). Chemical modification frequently disrupts this subunit communication and lowers *n*_H_: a comprehensive biophysical characterization of clinically relevant HBOCs reported *n*_H_ values of 2.46 for αα-cross-linked Hb, 1.56 for PEGylated Hb, and 1.00 for O-raffinose–polymerized Hb (Hemolink) (103). Because a reduced *n*_H_ flattens the dissociation curve toward a hyperbola, such HBOCs may load and release O_2_ less efficiently across the physiological PO_2_ gradient even when their P_50_ is appropriate.

In addition to affinity and cooperativity, the oxygen-binding behavior of cell-free Hb will also be affected by ambient temperature and pH through the Bohr effect (pH-dependent modulation of oxygen affinity) and Root effect (enhanced oxygen release at low pH) (109). Unlike native RBCs, where 2,3-diphosphoglycerate (2,3-DPG) allosterically lowers O_2_ affinity by stabilizing the T-state (110), most HBOC formulations lack this allosteric modulator. Therefore, deoxy Hb is mainly affected by the local pH gradient and the liquid-phase diffusion coefficient (98, 111). These characteristics together determine the oxygen exchange ability of HBOC in various physiological environments.

### 5.2 Oxygen diffusion and microvascular distribution

The physiological effect cannot be guaranteed by favorable oxygen-binding kinetics alone. The extent to which HBOCs can exert their oxygen-carrying role also depends on their distribution within the microvasculature and the efficiency of oxygen diffusion across the transport barrier that separates the carrier from the oxygen-consuming tissue. Diffusion limitations and the geometric structure of the microvascular network jointly determine the spatiotemporal profile of tissue oxygenation. The steepness of the local oxygen concentration gradient is the most critical determinant (112).

During natural transport, erythrocytes flow along the central streamline of the vessel, so that oxygen must first cross the RBC membrane and the surrounding cell-free layer (CFL) before reaching the endothelial and tissues (113). In contrast, cell-free HBOCs are evenly dispersed in the plasma, effectively eliminating these radial diffusion barriers. This accelerated O_2_ equilibration can promote oxygen release earlier in the precapillary arterioles, thereby steepening the longitudinal O_2_ gradient along the microvascular axis and potentially triggering autoregulatory vasoconstriction (37). Notably, this same property may facilitate O_2_ delivery to ischemic or hypoperfused tissues where RBC perfusion is impaired (114).

In the microenvironment of ischemic injury, severe trauma, or tumor hypoxic, normal RBC perfusion is blocked by vascular tortuosity, interstitial edema, and rheological disorders. In this case, the nanoscale size of HBOCs allows them to bypass spatial resistance, perfuse the blocked capillary network, and extend the functional Krogh tissue oxygen column (the radial tissue region oxygenated by each perfused capillary), thus reaching distant hypoxic regions (39).

These observations are consistent with microfluidic and computational evidence that tissue oxygenation is governed less by the static P_50_ than by the carrier's dynamic response to local flow fields and metabolic gradients (115). Thus, the physiological relevance of an HBOC hinges on whether oxygen unloading is appropriately matched to the local metabolic milieu in both space and time (116).

### 5.3 Vascular tone and nitric oxide bioavailability

Vascular tone dysregulation is the most important safety concern for HBOCs. Under normal conditions, cell-free Hb binds NO much more slowly than oxyhemoglobin does (14). A common mitigation strategy is to increase molecular size through PEGylation or polymerization, which reduces Hb extravasation into subendothelial spaces and thereby lowers NO consumption (117, 118). Nevertheless, even with this approach, systemic vascular resistance may still rise by 8–50 percent in animal models (119). Whether this leftover NO scavenging is safe depends on the patient. In young, healthy trauma patients with normal endothelial function, the body may compensate. In elderly patients with poor endothelial function, who are also the ones most likely to receive HBOCs during surgery, the same degree of NO scavenging could cause severe hypertension or myocardial ischemia. No clinical trial has grouped patients by baseline endothelial function, so this key safety question remains open.

### 5.4 Autoxidation to MetHb and ROS generation

Within systemic circulation, Hb in HBOCs can undergo autoxidation from Fe^2+^ to the inactive Fe³⁺ MetHb, resulting in the generation of ROS that impair endothelial integrity and compromise oxygen delivery (111, 120).

To reduce these risks, modern HBOC designs commonly incorporate antioxidant strategies (121), though the evidence supporting each approach varies considerably. The first strategy involves the use of small molecule antioxidants. For instance, vitamin C can rapidly donate electrons to neutralize free radicals (122). However, Edgworth et al. (123) found that antioxidants such as ascorbic acid alone had limited efficacy. In a kidney perfusion model, the addition of vitamin C to an HBOC-based perfusate did not improve organ quality compared with RBC-based perfusion. In contrast, Rerkshanandana et al. (124) demonstrated that combining vitamin C with antioxidant enzymes in modified Hb microparticles offered protective effects and stability under oxidative conditions. The second strategy employs nanozymes as an alternative to biological enzymes. Liu et al. (98) investigated the integration of gold-based nanozymes into MOF nanocarriers, leveraging their enzyme-like catalytic activity to scavenge ROS. Their findings revealed that this approach effectively inhibits Hb oxidation, thus providing a new strategy for developing novel artificial oxygen carriers with antioxidant protection.

### 5.5 Rheological and hemodynamic effects

In addition to biochemical compatibility, the performance and safety of HBOCs are determined by its hemodynamic and rheological behavior. At the microcirculatory level, oxygen transport is mechanistically coupled to blood viscosity, shear stress, and capillary recruitment (125, 126). Analytical modeling by Li et al. (125) demonstrates that the effect of increased blood viscosity on tissue oxygen delivery (DO_2_) is critically gated by the endothelium's capacity to respond to shear-mediated NO signaling. When endothelial responsiveness is intact, vasodilation overcomes the viscosity-induced flow hindrance. When it is absent, however, DO_2_ may paradoxically fall. This finding exposes a fundamental limitation in current HBOC design. Specifically, oxygen-carrying capacity alone is an insufficient target. The same formulation could improve DO_2_ in a patient with preserved endothelial function while worsening it in one with endothelial dysfunction due to age, disease, or hemorrhagic injury. Therefore, the long-pursued balance between oxygen delivery capacity and viscosity is not an inherent feature of this formulation. Rather, it is a product–host interaction that depends on the recipient's endothelial phenotype. This variable is almost never characterized in HBOC studies.

### 5.6 Interaction between immunity and complement

The clinical feasibility of HBOCs is largely determined by their immune tolerance. Unmodified or excessively cross-linked HBOCs may trigger complement activation and phagocytic clearance via the mononuclear phagocyte system (67, 68). Such events can precipitate complement activation-related pseudo-allergy (CARPA), which occurs independently of IgE-mediated mechanisms in susceptible individuals (68). To avoid immune recognition, surface modification with hydrophilic polymers or biomimetic coatings is commonly employed (127, 128). However, the immune response is not determined by surface chemistry alone. Particle size, surface charge, and surface curvature each govern the uptake of nanoparticles by macrophages. Yu et al. (129) demonstrated that PEGylated nanoparticles (30–100 nm) are internalized in a size-dependent manner via scavenger receptor A (CD204), and that particle size is a stronger determinant of uptake than charge. Charge exerts a non-monotonic effect, in which uptake is greatest for strongly positive or strongly negative surfaces and lowest at a weakly negative surface charge (approximately −9 to −3.5 mV). Surface curvature also shapes immune recognition. Because complement-fixing IgM binds membranes in a curvature-dependent manner, low-curvature micrometer-scale aggregates deposit more complement than high-curvature sub-100-nm vesicles, thereby promoting receptor-mediated phagocytosis (68). However, because size outweighs charge, weakly negatively charged Hb-loaded ZIF-8 nanoparticles (approximately 130–150 nm in diameter, above the 30–100 nm range tested) still adsorb opsonins such as IgG (21, 130) and would be expected to undergo mononuclear phagocyte clearance. Coating such particles with human serum albumin (130) or a PEGylated MPN (21) reduces this opsonin adsorption. Collectively, these observations indicate that immune compatibility requires a multi-parametric design rather than any single surface modification, to suppress complement activation, opsonization, and phagocytic clearance in concert.

## 6 Applications of translational medicine

HBOCs have evolved from experimental blood substitutes to oxygen therapy drugs that can be individually adjusted to specific clinical indications. Their clinic value is affected by many factors, such as the urgency of oxygen debt, the required support duration, the hemodynamic risk, and the feasibility of monitoring (131). Therefore, HBOCs should be evaluated according to established or selected clinical application scenarios, including trauma and hemorrhagic shock, perioperative oxygen support, military and prehospital first aid, organ preservation, and resource-limited environments (132). In these situations, the main clinical problem is not whether HBOCs can completely replace RBCs, but whether they can provide temporary oxygen-carrying support when routine blood transfusion is unavailable or inadequate (133, 134). Figure 3 summarizes the structural features of HBOCs, biological mechanisms, and the relationship among the proposed or emerging clinical application scenarios.

**Figure 3:**
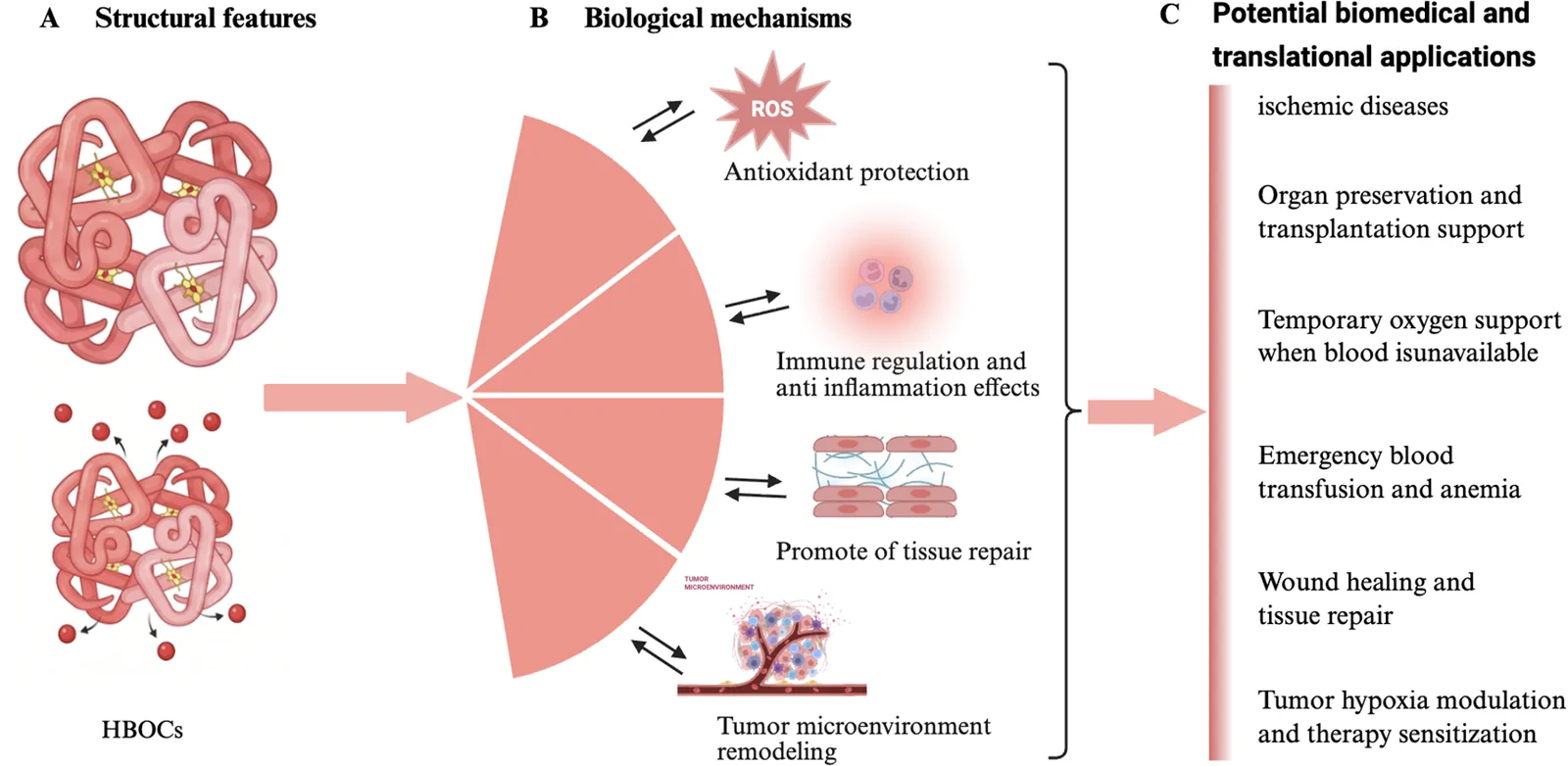
Overview of structural features, biological mechanisms, and potential biomedical and translational applications of HBOCs. Panel A shows the structural features of HBOCs, which can be engineered with tunable oxygen affinity to enhance oxygen transport and delivery. In addition to oxygen carriage, HBOC–based systems may contribute to antioxidant protection, immune regulation and anti–inflammatory effects, promotion of tissue repair, and tumor microenvironment remodeling. The biological mechanisms shown in Panel B include clinically explored and preclinical, depending on the specific HBOC formulation, disease model, and application context. The applications listed in Panel C represent potential biomedical and translational uses.

### 6.1 Trauma and hemorrhagic shock

Trauma and hemorrhagic shock are among the most direct yet challenging indications for HBOCs (135). In these scenarios, the therapeutic objective is to rapidly restore the oxygen-carrying capacity of plasma until compatible RBCs become available (136, 137).

Compared with crystalloids (electrolyte solutions such as normal saline or lactated Ringer's) or colloids (solutions of larger molecules such as albumin or hydroxyethyl starch), HBOCs have two main therapeutic advantages: blood volume expansion and oxygen transport (26). Blood volume expansion increases circulating volume and perfusion pressure, thereby maintaining systemic hemodynamics. Effective oxygen delivery depends on Hb concentration, oxygen affinity, microvascular flow, and tissue oxygen unloading (102–104, 138). In a rat hemorrhagic shock model, Williams et al. (139) demonstrated that high-molecular-weight PolybHb restored cardiac function as effectively as fresh whole blood, and its safety and efficacy remained unchanged after two years of storage. These findings indicate that, under controlled conditions, optimized HBOC formulations can adequately preserve the key properties that govern oxygen delivery. In contrast, blood stored for 3 weeks required a larger transfusion volume and was associated with elevated markers of organ damage, highlighting that even RBC-based transfusion can fail when oxygen affinity and microvascular delivery are compromised by storage lesions. In a multicenter phase III trial assessing survival of patients resuscitated with an HBOC initiated at the scene of injury, Moore et al. (50) found no significant difference in 30-day mortality between the PolyHeme and control groups, yet adverse events were more frequent in the PolyHeme group.

Trauma patients often have acidosis, coagulopathy, and endothelial injury. These conditions may worsen several HBOC-related side effects, including vasoconstriction, hypertension, MetHb formation, and oxidative stress (111, 140). As a result, effective oxygen delivery can be undermined by the very pathological state that HBOCs are intended to treat. HBOCs should therefore be viewed as investigational, short-term bridge therapies in trauma and hemorrhagic shock, not as definitive resuscitation fluids or complete blood substitutes.

### 6.2 Perioperative and surgical oxygen support

Compared with unpredictable trauma, the perioperative period and the surgical environment may provide more controllable translational research settings. When there is a need to conserve blood, when compatible blood is unavailable, or when the patient refuses allogeneic transfusion, the oxygen carrier can be regarded as a research aid (141).

In surgery, HBOCs can be used as a temporary oxygen carrier during acute anemia or as an auxiliary means of the patient's blood management plan; however, their use is still highly dependent on the specific situation and does not replace conventional transfusion (142). Patients need to be carefully selected for perioperative application. Patients with active myocardial ischemia, severe coronary artery disease, pulmonary hypertension, uncontrolled hypertension, advanced renal failure, or increased risk of thrombosis may be particularly susceptible to HBOC-related vasoactivity and oxidative damage (11, 64, 143). Therefore, use in surgery should be limited to patients with a favorable benefit-to-risk ratio, in settings with regulatory approval or appropriate research programs, and with sufficient monitoring capabilities (47).

### 6.3 Military medicine and resource-limited environment

In military and resource-limited settings, the logistical advantages of HBOC are particularly significant. HBOC can be formulated as a preparation with a long shelf life, which can be deployed quickly and used without blood typing or crossmatching (144). The aging donor population and the shrinking pool of eligible donors have exposed the fragility of the blood supply (145). This vulnerability becomes especially acute during natural disasters and military conflicts. These findings highlight the potential of transfusion alternatives to meet both current and future medical needs.

However, several obstacles still hinder the practical application of HBOCs. First, the stability of HBOCs under extreme environmental conditions, such as temperature fluctuations, elevated humidity, and mechanical vibration, has not been verified on a large scale. Second, owing to the complexity of Hb purification, chemical cross-linking, and encapsulation processes and the demands of Good Manufacturing Practice (GMP) standards, their production cost remains far higher than that of donated blood (146). Third, the regulatory pathway for HBOCs is extremely stringent: given both their novelty as oxygen-carrying biological products and the safety concerns raised in early clinical trials, they face more rigorous evidence requirements from regulatory agencies such as the FDA (147). Whether these barriers can be overcome, and whether the risk–benefit balance of HBOCs in combat casualties is favorable, remain open questions.

### 6.4 Organ preservation and transplantation

Organ preservation is one of the most clinically feasible applications for HBOCs. Because exposure is primarily confined to the *ex vivo* perfusion circuit or graft environment, the risks associated with systemic infusion are substantially reduced, including hypertension, systemic vasoconstriction, and widespread immune activation (64). Beyond this favorable safety profile, the oxygen-carrying capacity of HBOCs may directly support graft viability, making them particularly attractive candidates in this setting (148, 149).

In the OXYgen carrier for Organ Preservation (OXYOP) multicenter open-label study conducted across six transplant centers in France. Meur et al. (150) evaluated the safety of M101, as an additive to kidney preservation solution in human renal transplantation. Sixty grafts received M101, with the contralateral kidney serving as a paired control. M101 demonstrated a favorable safety profile with no allergic or immune reactions. Analysis of secondary endpoints revealed significantly lower rates of delayed graft function. In addition, during hypothermic or normothermic machine perfusion, HBOCs that enhance oxygen diffusion, preserve mitochondrial function, and reduce ischemia-reperfusion have been evaluated injury in organ preservation models (151). Laing et al. (152) conducted the first evaluation of HBOC-201 in normothermic machine perfusion of human liver grafts. Perfusates based on HBOC and on RBCs achieved comparable vascular flow, lactate clearance, and histological integrity. The HBOC group also showed higher oxygen extraction, with no evidence of cytotoxicity or histological damage *in vitro*.

These findings suggest that restricting HBOC exposure to the *ex vivo* organ, rather than the systemic circulation, can avoid systemic toxicity while providing oxygenation benefits. Organ preservation may thus represent a favorable near-term indication for the clinical translation of HBOCs. Table 2 summarizes the clinical and translational evidence for representative HBOCs evaluated in trauma, perioperative, military, and organ-preservation settings, organized by design, intended indication, evidence type, main findings, and current regulatory or translational status.

**Table 2:** Clinical and translational evidence for representative HBOCs.

Product	Design	Indication	Evidence	Main findings	Status	Ref
DCLHb/HemAssist	Human Hb; diaspirin cross-linked	Trauma; hemorrhagic shock	Phase II/III trials	No consistent survival benefit; ↑mortality, hypertension, ↑CV risk	Discontinued;	(23, 153)
PolyHeme	Human Hb; glutaraldehyde-polymerized	Prehospital trauma	Phase III trial	Non-inferiority not met; ↑CV events; EFIC concerns	Discontinued;	(11, 36)
HBOC-201/Hemopure	Bovine Hb; glutaraldehyde-polymerized	Oxygen bridge (RBC unavailable/contraindicated)	Clinical/compassionate use	Bridge benefit (selected cases); ↑MetHb, hypertension, ↑CV risk	Approved (South Africa)	(154, 155)
Hemolink	Human Hb; O-raffinose cross-linked	CABG; perioperative O_2_ support	Phase II/IIIl trials	Limited efficacy; hemodynamic instability; poor tolerability	Discontinued	(156, 157)
MP4OX/Hemospan	Human Hb; PEGylated	Perioperative ischemia	Phase II/III evaluation	Microvascular O_2_ benefit; uncertain efficacy; hemodynamic concerns	Discontinued	(25, 158)
SANGUINATE	Bovine Hb; PEGylated carboxyhemoglobin	Sickle cell disease; ischemia	Early clinical studies	Improved O_2_ transfer; limited safety data; ↑MetHb	Investigational	(159, 160)
HbV	Human Hb; lipid encapsulation	Hemorrhagic shock; organ protection	Advanced animal/NHP studies	↓Vasoconstriction vs. acellular Hb; complement/RES challenges	Advanced preclinical	(61, 161)
M101/HEMO2life	Marine Hb (*Arenicola marina)*	Organ preservation; transplantation	Clinical evaluation	Improved graft preservation; immunogenicity; limited systemic safety data	Clinical (organ preservation only)	(73, 150)
Recombinant Hb	Genetically engineered Hb	Tunable O_2_ therapeutics	Preclinical/early translational	Rational O_2_-affinity design; reduced NO reactivity; manufacturing/safety barriers	Preclinical	(49, 90)

Hb, hemoglobin; HBOC, hemoglobin-based oxygen carrier; NO, nitric oxide; MetHb, methemoglobin; O2, oxygen; CABG, coronary artery bypass grafting; PEG, polyethylene glycol; RES, reticuloendothelial system; RBC, red blood cell; CV risk, cardiovascular risk; NHP, Non-human primate; EFIC, exception from informed consent.

### 6.5 Localized hypoxia-related microenvironments and bioengineered hemoglobin platforms

Beyond systemic oxygen delivery, Hb has been integrated into bioengineering platforms to address a range of localized hypoxia-related conditions. Here, we use the term “Hb-based platforms” broadly to encompass both classical HBOCs and newer engineered formulations such as conjugates, vesicles, composites, and hydrogels, the former developed primarily for intravascular oxygen transport in blood-substitute applications. These formulations are designed for localized oxygen delivery and, in some cases, for the co-delivery of drugs or therapeutic gases and the modulation of immune responses. Four representative applications are discussed below: tumor therapy, antibacterial therapy, anti-inflammatory therapy, and wound healing. An overview of these four applications and their representative Hb-based platforms is shown in Figure 4.

**Figure 4:**
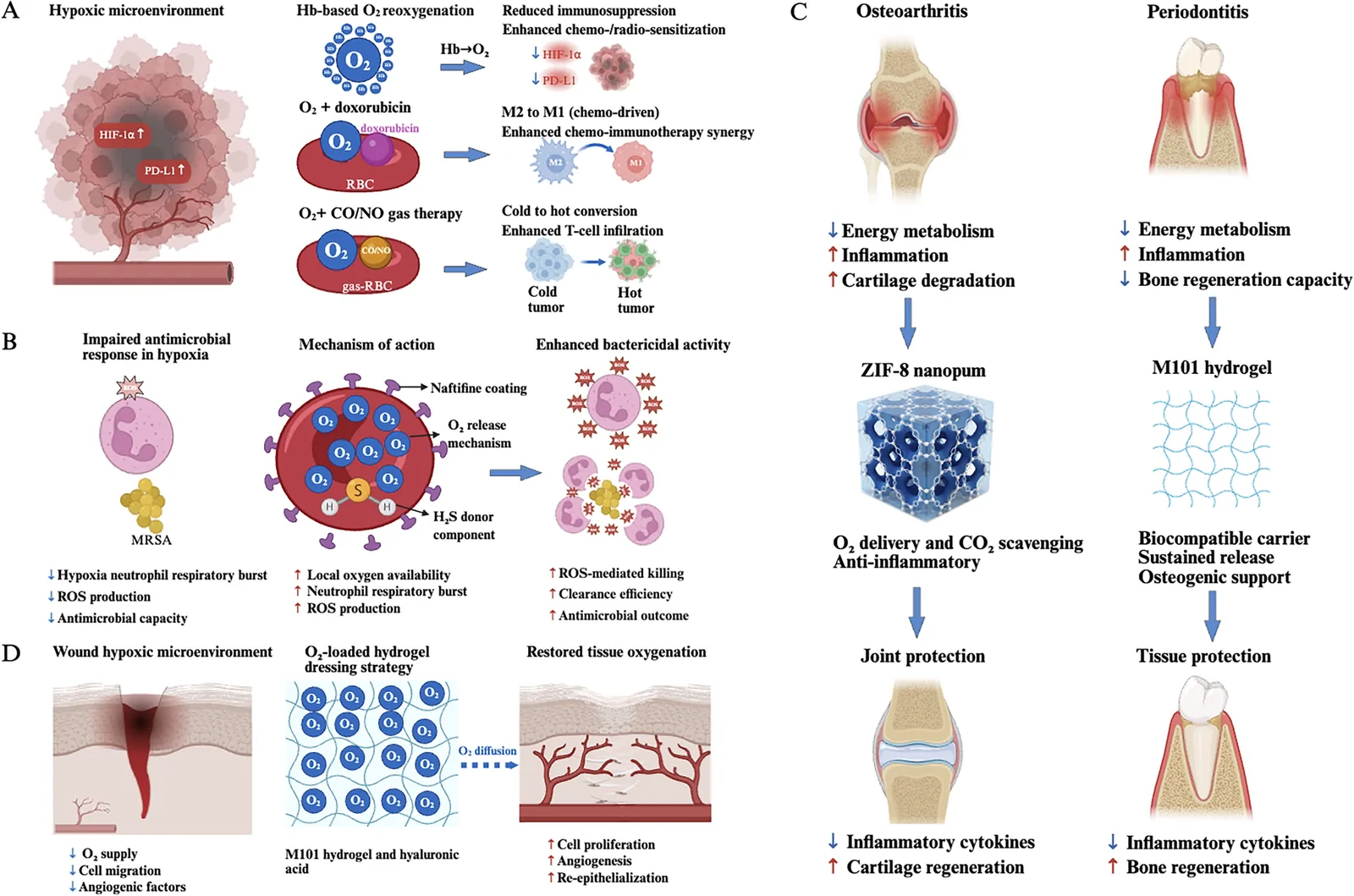
Bioengineered oxygen platforms for localized hypoxia-related diseases. Schematic illustration of bioengineered oxygen platforms that restore local oxygen homeostasis and reverse hypoxic pathophysiology across multiple disease contexts. **(A)** In solid tumors, multiple oxygen-delivery strategies synergistically enhance chemo-/immunotherapy sensitivity by modulating the tumor microenvironment. **(B)** In bacterial infections, ZIF-8-based nanoplatforms with H_2_S donors restore neutrophil respiratory burst and antimicrobial capacity. **(C)** In chronic inflammatory diseases (osteoarthritis and periodontitis), ZIF-8 nanoparticles and M101 hydrogel reduce inflammation while promoting cartilage/bone regeneration. **(D)** In wound healing, O_2_-loaded hydrogels facilitate angiogenesis and re-epithelialization.

#### 6.5.1 Tumor therapy

Hypoxia is a hallmark of solid tumors (162). Hypoxia can stabilize hypoxia-inducible factor-1α (HIF-1α) and promote programmed death-ligand 1 (PD-L1) expression in tumor and myeloid cells, while favoring an immunosuppressive microenvironment that includes M2-like tumor-associated macrophages (TAMs) (163, 164). Together, these changes are associated with chemoresistance, immune evasion, and metastasis (165). Hb-based nanoplatforms have therefore been designed to relieve tumor hypoxia in order to sensitize tumors to chemotherapy and immunotherapy.

Wang et al. engineered a biomimetic nano-RBC by conjugating Hb to a polymer and loading doxorubicin (166). Hb on the carrier bound plasma haptoglobin, and the resulting haptoglobin-Hb complex was recognized by CD163-expressing M2 macrophages, enabling preferential uptake and acid-responsive doxorubicin release. The delivered oxygen downregulated HIF-1α/PD-L1 and reshaped the immunosuppressive microenvironment, markedly improving survival in a colorectal model. Li et al. developed gas-loaded nano-RBCs that release carbon monoxide (CO) and NO in the tumor milieu (167). These carriers disrupt glycolysis and convert immunologically “cold” tumors into “hot” ones, nearly eradicating lung metastases when combined with anti-PD-L1. Earlier, Hb-loaded nanoliposomes alleviated tumor hypoxia by reducing HIF-1α and vascular endothelial growth factor (VEGF) without organ toxicity (168). Collectively, these platforms show how Hb-based carriers combine oxygen, drug, and gas delivery with immunomodulation to broaden cancer therapy.

#### 6.5.2 Antibacterial therapy

Hypoxia impairs the neutrophil respiratory burst by limiting oxygen availability for NADPH oxidase-mediated ROS production. This reduction in ROS generation directly compromises the oxidative killing capacity of neutrophils, thereby weakening antimicrobial defense (169). Zhu et al. combined Hb with naftifine and a RBC-membrane coating to create a nanoimmunotherapy that engages neutrophils against *Staphylococcus aureus* (*S. aureus*) (170). Naftifine blocks staphyloxanthin, the pigment that shields the pathogen from oxidative killing. Hb then catalyzes the oxidation of bacterial hydrogen sulfide, generates neutrophil-chemoattractant lipid products, and supplies oxygen to restore neutrophil respiratory burst under hypoxia. This platform achieved over 99.9% bacterial clearance in mouse models of methicillin-resistant *S. aureus* (MRSA) thigh infection, pneumonia, and peritonitis, as well as multidrug-resistant *S. aureus* bacteremia. In the lethal peritonitis and bacteremia models, treatment with the platform achieved 100% survival at 14 days, vs. 12.5% for free naftifine and 37.5% for the same nanoparticles without the RBC membrane coating. These results were obtained in single experiments with 6–8 mice per group, and no resistance emerged after ten serial passages *in vitro*. However, this short-term assay does not exclude resistance development under clinically relevant selective pressures. Although promising, these findings remain limited to acute mouse infection models and have yet to be validated in humans. Hb-mediated redox chemistry may also generate oxidizing intermediates and promote heme- or iron-associated tissue injury when inadequately controlled; this safety liability should be considered across Hb-based antibacterial platforms and other locally applied therapeutics (29).

#### 6.5.3 Anti-inflammatory therapy

Oxygen carriers have also been repurposed to counteract inflammation in two representative settings.

The first setting is osteoarthritis. In osteoarthritic cartilage, low oxygen tension limits aerobic energy production and repair. Xia et al. (171) developed a Hb-loaded ZIF-8 “nanopump” with a cartilage-targeting peptide. This nanopump disassembles in response to ultrasound and the acidic cartilage microenvironment, releasing oxygen and adsorbing carbon dioxide to reactivate energy metabolism. In a post-traumatic osteoarthritis model, it mitigated cartilage degeneration, subchondral bone sclerosis, and synovial inflammation while improving motor function.

The second is periodontitis. M101 is a natural extracellular Hb of the marine lugworm *Arenicola marina*, with intrinsic antioxidant activity and low immunogenicity. In epithelial cells, M101 reduced inflammation induced by *Porphyromonas gingivalis* (*P. gingivalis*), downregulating tumor necrosis factor-α (TNF-α), nuclear factor-κB (NF-κB), and receptor activator of nuclear factor-κB ligand (RANKL) while upregulating pro-healing mediators. It also reduced *P. gingivalis* biofilm growth *in vitro*, and in a mouse calvarial abscess model, decreased abscess size and inflammatory score (172). In a separate mouse periodontal-pocket model, the hydrogel formulation HEMDental-Care improved epithelial and fibrous attachment and increased bone gain (173). These periodontal results remain preclinical, as a human trial is still in preparation.

#### 6.5.4 Wound healing

Wound hypoxia impedes cell proliferation, angiogenesis, and re-epithelialization, while also compromising immune function. It is therefore considered a key driver of chronic non-healing wounds (174). HBOCs have thus been embedded in dressings to deliver oxygen locally and in a controlled manner. Han and colleagues reviewed several such platforms in the literature, including a black-phosphorus Hb microneedle with near-infrared-responsive oxygen release, a light-responsive oxyhemoglobin hydrogel, and a silk fibroin Hb gallium hydrogel that both oxygenates the wound and exerts antibacterial activity, achieving near-complete closure of infected diabetic wounds (174). Building on the same principle, HEMHealing, a hydrogel of M101 with hyaluronic acid, delivers oxygen passively along a partial-pressure gradient. In an *in vitro* wound-scratch assay, this hydrogel achieved complete closure within 24 h (173). Because M101 releases oxygen in a regulated, gradient-driven manner, it is under evaluation for chronic and ischemic wound repair, although its clinical efficacy remains to be established.

However, these applications remain largely experimental. The main obstacles involve long-term biological distribution, nanotoxicity, sterilization process, and immune clearance (175, 176). More broadly, these difficulties reveal a recurring problem in the field of HBOC: clinical value is likely to depend on strictly defined indications, prudent selection of patients or application scenarios, and a favorable balance between the benefits of oxygen supply and drug-related risks. Table 3 summarizes representative Hb-based platforms across these applications, organized by composition, target indication, intended application, and key function.

**Table 3:** Representative HBOCs and hb-containing nanoplatforms investigated for localized hypoxia-related diseases.

Platform composition	Targeted disease/indication	Intended application	Key functions	Ref.
HBOCs				
Polymerized human Hb	Solid tumors	Tumor oxygenation to sensitize radio/chemotherapy	Modulate tumor oxygenation; decrease hypoxia-driven resistance	(102, 126)
HBOC-201	Ischemic limb/chronic wound	Local O_2_ delivery beyond anemia bridge	Restore tissue oxygenation	(134)
M101-hydrogel	Periodontitis	Anti-inflammatory and tissue repair	Decrease TNF-α/NF-κB/RANKL; increase attachment, bone gain	(172, 173)
M101-HA hydrogel	Chronic wounds	Wound healing	Gradient-driven O_2_ delivery	(173)
Nanoplatforms				
Hb–polymer+doxorubicin (nano-RBC)	Colorectal cancer	Tumor oxygenation and chemotherapy	Relieve hypoxia; decrease HIF-1α/PD-L1; reprogram M2 TAM	(166)
Gas-loaded nano-RBC (CO/NO)	Lung metastasis	Gas therapy combined with immunotherapy	Transform cold tumors into hot tumors; disrupt glycolysis	(167)
Hb-loaded nanoliposomes	Solid tumors	Tumor oxygenation	Reduce HIF-1α and VEGF signaling	(168)
Hb + naftifine (RBC membrane)	MRSA/pneumonia/peritonitis	Antibacterial nanoimmunotherapy	Restore neutrophil burst	(170)
Hb@ZIF-8 nanopump	Osteoarthritis	Anti-inflammatory	ultrasound/pH-triggered O_2_ release and CO_2_ adsorption	(171)

Hb, hemoglobin; HBOCs, hemoglobin-based oxygen carriers; O_2_, oxygen; TNF-α, tumor necrosis factor-α; NF-κB, nuclear factor κB; RANKL, receptor activator of nuclear factor κB ligand; HIF-1α, hypoxia-inducible factor-1α; PD-L1, programmed death ligand-1; M2 TAM, M2 tumor-associated macrophage; CO, carbon monoxide; NO, nitric oxide; VEGF, vascular endothelial growth factor; MRSA, methicillin-resistant Staphylococcus aureus; RBC, red blood cell; pH, potential of hydrogen; CO_2_, carbon dioxide.

## 7 Safety evaluation: pharmacokinetics, toxicology, and immunogenicity

The clinical application of HBOCs depends on their oxygen delivery efficiency as well as complex pharmacological and safety factors that affect clinical outcomes and regulatory approval. Despite extensive research, the clinical translation of HBOCs remains limited by a narrow therapeutic range, multiple safety concerns, and challenges in large-scale production and ethical concerns (26, 78, 147).

### 7.1 Pharmacokinetics, biodistribution, and elimination mechanisms

HBOCs show formulation-dependent pharmacokinetics, with circulation half-life in humans ranging from roughly 2–24 h, varying with molecular size, degree of polymerization, and surface modification (74, 160). Under physiological conditions, native cell-free Hb is scavenged by haptoglobin, which binds Hb dimers and delivers them via CD163 to hepatic and splenic macrophages, whereas free heme is bound by hemopexin and delivered via CD91 predominantly to hepatocytes (177). Renal filtration becomes significant only when this haptoglobin capacity is exceeded and the 64 kDa tetramer dissociates into filterable 32 kDa αβ dimers (178). In contrast, chemical modification prevents the formation of the αβ dimers that haptoglobin requires, so HBOCs largely bypass this scavenging system and are instead cleared by size-dependent routes (177, 179). Intramolecular cross-linking that blocks tetramer dissociation, or polymerization and PEGylation that enlarge the hydrodynamic radius, therefore reduce glomerular filtration and prolong retention (180). Two routes then dominate: unmodified or small species are filtered and reabsorbed in the proximal tubule via megalin and cubilin (181), whereas larger species that escape filtration are taken up by MPS macrophages of the liver and spleen, a route best characterized for particulate carriers such as HbVs (67). Because free tetramer content correlates with nephrotoxicity, minimizing residual unmodified hemoglobin is a key quality-control consideration (182).

Within macrophages, Hb is degraded and the liberated heme is cleaved by heme oxygenase-1 (HO-1) to biliverdin, CO, and Fe^2+^. Biliverdin is reduced to bilirubin and excreted in bile, CO is eliminated in exhaled air, and most iron is exported via ferroportin and recycled to erythropoiesis, with ferritin buffering the labile pool (183). Since the Nrf2–HO-1–ferritin axis has finite, saturable capacity (184), sustained heme loading raises hepcidin, which restricts ferroportin-mediated export and thereby retains iron within hepatic and splenic macrophages, contributing to oxidative injury (185, 186). Controlling molecular size, surface chemistry, and residual free tetramer is therefore central to balanced oxygen delivery, efficient clearance, and limited toxicity.

### 7.2 Toxicological and hemodynamic considerations

The main clinical limitations of HBOCs come from systemic and microcirculatory disorders related to the physicochemical properties of acellular Hb (140). The removal of NO leads to vasoconstriction and transient hypertension, while the autoxidation of heme iron will produce MetHb and ROS, both of which reduce oxygen-carrying capacity and increase oxidative damage (111). In addition, glomerular filtration of Hb dimers and subsequent heme deposition may lead to renal tubular toxicity (187).

To alleviate these adverse reactions, a variety of optimization strategies have been extensively studied. These methods include molecular shielding modification (such as PEGylation or liposome encapsulation), the combined use of vasodilators or antioxidants, and strict hemodynamic monitoring during infusion (188). Additionally, in specific clinical situations, HBOCs can provide clinical benefit with an acceptable safety profile. Colin et al. (52) showed that early expanded access use of HBOC-201 in patients with Hb below 5 g/dL optimized survival and reduced advanced resource utilization. In Phase III trials, transfusion was avoided in 96% of patients within 24 h and in 70% within one week. Patients who received 10 or fewer bags of HBOC-201 and those who received 3 or fewer units of blood showed no significant difference in serious adverse events or mortality. However, non-serious adverse events were more frequent in the HBOC-201 group. Overall, these strategies emphasize the need for an integrated management framework to minimize side effects while maintaining therapeutic efficacy.

### 7.3 Immunogenicity and inflammatory responses

Despite the lack of native cell membrane structure, HBOCs can still trigger innate immune responses through interaction with plasma proteins, complement components, and endothelial receptors (189). Therefore, immunotoxicity evaluation must clearly consider CARPA, a pseudoallergic reaction mediated by complement activation (68). In addition, circulating HBOCs are processed by the reticuloendothelial system (RES), in which macrophage uptake, processing, and metabolism affect clearance kinetics and immune outcomes (67). Therefore, comprehensive immunotoxicity assessment is a key link in regulatory evaluation and should include cytokine profiling, macrophage activation and phagocytosis assays, and endothelial barrier integrity assays (190).

Recent translational studies have shown that strategies such as surface PEGylation, antioxidant co-formulation, and controllable redox regulation can reduce cytokine release, weaken complement activation, and improve overall biocompatibility in preclinical and translational studies (191). When a PEGylated surface is used, immunogenicity evaluation must specifically target anti-PEG immune responses. Preclinical data show that repeated administration of PEGylated liposomes may induce accelerated blood clearance (ABC), which has been linked to anti-PEG immunoglobulin M (IgM) elicited by subsequent dosing and to complement activation (65). A minimal physiologically based pharmacokinetic (PBPK) framework further suggests that anti-PEG antibody levels, particularly immunoglobulin G (IgG)-related clearance dynamics, quantitatively influence the loss of prolonged circulation of PEGylated therapeutics upon re-dosing (192). Therefore, repeated-dose safety should be assessed using product-specific dosing regimens and evidence-based immunological endpoints, including complement activation and inflammatory markers. In the presence of PEG, monitoring anti-PEG and ABC-related immune responses is essential.

## 8 Challenges, limitations, and future research priorities

### 8.1 Toxicities and knowledge gaps

Experience with HBOC development has demonstrated that oxygen-carrying capacity alone is insufficient to predict clinical benefit. Although early preparations have confirmed the feasibility of plasma-based oxygen transport and, in certain clinical settings, reduced the need for allogeneic blood transfusion, these advantages have often been offset by preparation-specific toxicities (37, 50). The most prominent of these are NO scavenging, vasoconstriction, oxidative stress, and endothelial dysfunction. Therefore, HBOCs should not be positioned as universal RBC substitutes, but as experimental oxygen therapeutics tailored to specific conditions where temporary oxygen support would offer clear benefits over existing alternatives (193). Beyond these safety concerns, several knowledge gaps continue to impede clinical translation. First, the quantitative relationship between oxygen affinity, redox behavior, microvascular perfusion, and tissue oxygenation has not been systematically elucidated (24, 25, 191). Second, validated biomarkers for predicting efficacy or toxicity remain lacking, and existing preclinical models cannot fully replicate human pathophysiology. Third, for emerging platforms such as Hb-containing nanoparticles and bioengineered constructs, systematic evaluation of their long-term performance remains incomplete (176, 194). Finally, owing to substantial differences in study design, synthesis of clinical evidence remains difficult (141).

### 8.2 Manufacturing, sourcing, shelf life and cost considerations

Beyond these biological obstacles, differences in sourcing, manufacturing, shelf life, and cost exist across different generations and platforms of HBOCs.

First- and second-generation products were derived from outdated donated human blood, such as SFH, DCLHb, and PolyHeme, or from bovine blood, as in the case of HBOC-201 (10). The supply of human blood is inherently limited, which is precisely one of the challenges that HBOCs are intended to address. Although bovine blood is more abundant, its use requires closed herds, validated viral-clearance procedures, and rigorous control of transmissible spongiform encephalopathy (TSE) risks (105).

Manufacturing complexity has increased across generations, progressing from stroma removal and cross-linking to glutaraldehyde polymerization, PEGylation, and liposomal encapsulation (37). The large-scale manufacture of these products, particularly encapsulated formulations, remains challenging (39). Recombinant Hb eliminates dependence on donor blood and reduces the risk of pathogen transmission but is constrained by low functional yields and stringent endotoxin-control requirements (49).

The storage requirements of HBOCs differ substantially from those of RBCs. Refrigerated RBCs can be stored for several weeks, depending on the additive solution used (195). In contrast, polymerized HBOCs such as HBOC-201 and PolyHeme can be stored at ambient temperature for extended periods without requiring cross-matching, representing an important logistical advantage over RBCs. This stability is achieved through deoxygenated or carboxy-liganded formulations and oxygen-impermeable packaging, which limit MetHb accumulation during storage (52). Stability data for encapsulated platforms remain comparatively limited, and vesicle fusion or lipid oxidation may occur during storage (161).

Manufacturing costs are influenced by production scale, process complexity, and quality-control requirements. Purification, pathogen removal, and endotoxin clearance can contribute substantially to overall production costs, particularly for formulations requiring multiple processing steps (26).

Accordingly, these constraints make HBOCs unsuitable as a general substitute for donated blood. Their use may instead be most valuable in settings such as prehospital resuscitation, military care, transfusion refusal, and regions with limited access to a safe blood supply.

### 8.3 Ethical and trial-design limitations

A further limitation concerns the ethical dilemma inherent in studying the population most likely to receive HBOCs. HBOCs have known toxicities, so their use may be justified mainly in patients who lack an acceptable alternative to transfusion, such as those who decline transfusion for religious reasons, cannot be cross-matched, or are treated in settings where blood is unavailable. Yet, these are largely the same patients who cannot be enrolled in trials that randomize HBOCs against standard transfusion, because they decline the control intervention. As a review of transfusion-refusing patients states, their refusal “means they would not participate in the pivotal clinical trials comparing haemoglobin-based oxygen carriers or alternatives to standard of care” (196). Evidence in this population therefore comes mostly from case reports and compassionate use, such as PolyHeme given to patients who declined transfusion for religious reasons, and this early evidence has not been confirmed in later comparative studies. Randomization against transfusion is also ethically sensitive: in the PolyHeme trial, the product was continued even when blood became available, which drew criticism because a patient with an available standard treatment is generally entitled to receive it (197). These constraints do not preclude generating evidence, but they change the kind of evidence that can be obtained. These patients can still be studied through designs that do not require a transfusion control arm, such as prospective cohort and registry-based studies, target-trial emulation, and historical or external controls. Organ preservation offers a comparator independent of systemic transfusion, such as a paired contralateral organ (150), and expanded-access data, including the 1,701 patients who received HBOC-201 (52), provide real-world evidence for patients with no transfusion alternative.

### 8.4 Future research priorities

Future research should be guided by mechanistic understanding and tailored to specific indications (10, 47). At the molecular design level, priority should be given to mitigating oxidative damage, optimizing oxygen release kinetics, and enhancing biocompatibility. In preclinical evaluation, researchers should adopt clinically relevant models that assess microcirculatory function, endothelial integrity, coagulation status, and long-term safety. At the clinical level, evaluation should proceed cautiously, and only after preclinical safety has been demonstrated *in vitro*, including in organoid models, and in appropriate animal models, beginning with early-phase human studies in narrowly defined, high-need populations, using clinically meaningful safety endpoints. Ultimately, successful clinical translation of HBOCs will depend on mechanistically informed design and well-defined indications. It will also require effective oxygen delivery and practicality in sourcing, manufacturing, storage, and cost.

Finally, the field should avoid characterizing each new platform from scratch. Despite differences in toxicity profiles and dose requirements, cell-free Hb products are associated with several common safety concerns, including NO scavenging, oxidative stress, and endothelial dysfunction. Standardized preclinical assays, biomarkers, and reference comparators would enable cross-platform comparisons and evidence accumulation. Where feasible, new platforms could be developed through incremental modifications of established scaffolds. Evaluation could then emphasize the modified features while ensuring appropriate characterization of the complete product. Shared preclinical datasets, standardized reporting, and registry-based clinical evidence could further reduce duplication, development time, and cost.

## 9 Conclusions

HBOC research has progressed through several distinct stages: the early pursuit of a universal blood substitute, the recognition of the fundamental toxicity of cell-free Hb following clinical trial failures, and the current era of mechanism-driven molecular design tailored to specific indications. On balance, the evidence supports cautious optimism. The field has demonstrated that cell-free Hb can effectively transport oxygen; doing so safely in compromised patients remains the challenge. The “oxygen bridge” concept reframes the clinical value proposition from replacement to temporary support.

The early evidence from M101 in *ex vivo* organ preservation demonstrates that HBOCs can provide clinical benefit when systemic exposure is minimized. Although the core challenges identified in first-generation clinical trials are now better understood, they remain unsolved for systemic indications. These challenges stem from the narrow and unpredictable therapeutic window caused by NO scavenging, oxidative stress, and immune activation. Overcoming them will require both improved molecular designs and stronger supporting evidence, including safety biomarkers validated through prospective studies, reproducible large animal experiments from independent laboratories, and cautiously staged early-phase studies preceded by rigorous preclinical safety testing. For the no-alternative indications, registry, expanded-access, and observational evidence will likely have to supply the comparative data that randomization cannot provide. The field is progressing toward a portfolio of indication-specific oxygen therapeutics that is promising in concept but largely unproven in practice. Realizing its potential will demand sustained interdisciplinary collaboration, a willingness to abandon approaches that fail rigorous testing, and an insistence on the same rigor before any new platform reaches the clinic.

## Glossary

RBC: Red blood cell
HBOC: Hemoglobin-based oxygen carrier
FDA: Food and Drug Administration
Hb: Hemoglobin
NO: Nitric oxide
MetHb: Methemoglobin
MPN: Metal-phenolic network
β-F41K: β-chain phenylalanine-to-lysine substitution at position 41
ZIF-8: Zeolitic imidazolate framework-8
PEG: Polyethylene glycol
PFC: Perfluorocarbon
SFH: Stroma-free hemoglobin
DCLHb: Diaspirin cross-linked hemoglobin (HemAssist)
JAMA: Journal of the American Medical Association
PolybHb: Polymerized bovine hemoglobin
RR: Risk ratio
ROS: Reactive oxygen species
PEG-Hb: PEGylated hemoglobin
HbV: Hemoglobin vesicle
LEH: Liposome-encapsulated hemoglobin
MPS: Mononuclear phagocyte system
P_50_: Oxygen partial pressure at which hemoglobin is 50% saturated
PDA: Polydopamine
SOD: Superoxide dismutase
Hb@MOF: Hemoglobin-encapsulated metal-organic framework
MOF: Metal-organic framework
ZIF: Zeolitic imidazolate framework
*n*_H_: Hill coefficient
2,3-DPG: 2,3-diphosphoglycerate
CFL: Cell-free layer
DO_2_: Tissue oxygen delivery
CARPA: Complement activation-related pseudoallergy
IgE: Immunoglobulin E
CD204: Scavenger receptor A
IgM: Immunoglobulin M
IgG: Immunoglobulin G
GMP: Good manufacturing practice
OXYOP: Oxygen carrier for organ preservation
HIF-1α: Hypoxia-inducible factor-1α
PD-L1: Programmed death-ligand 1
TAM: Tumor-associated macrophage
CO: Carbon monoxide
VEGF: Vascular endothelial growth factor
S. aureus: Staphylococcus aureus
MRSA: Methicillin-resistant staphylococcus aureus
TNF-α: Tumor necrosis factor-α
NF-κB: Nuclear factor-κB
RANKL: Receptor activator of nuclear factor-κB ligand
HO-1: Heme oxygenase-1
RES: Reticuloendothelial system
ABC: Accelerated blood clearance
PBPK: Physiologically based pharmacokinetic
TSE: Transmissible spongiform encephalopathy.
